# Ataxia associated with an interhemispheric subdural hematoma: a case report

**DOI:** 10.4076/1757-1626-2-8876

**Published:** 2009-08-12

**Authors:** Johanna C Schilder, Martijn Weisfelt

**Affiliations:** Department of Neurology, Kennemer GasthuisBoerhaavelaan 22, 2035 RC, HaarlemThe Netherlands

## Abstract

Interhemispheric subdural hematomas are uncommon lesions. This case report describes a 77-year-old woman using anticoagulants who suddenly developed headache and ataxia of both legs. Computed tomography of the brain revealed an interhemispheric subdural hematoma, which was treated conservatively. Interhemispheric subdural hematomas should be considered in patients, especially in those using anticoagulants, even in the absence of trauma.

## Introduction

The exact incidence of interhemispheric subdural hematomas (ISH) is unknown. Only about 100 cases have been described in detail since the first description by Aring and Evans in 1940 [1].

The generally accepted causative mechanism for the development of ISH is tearing of the fixed bridging veins between the medial cerebral cortex and the superior sagittal sinus. ISH are associated with trauma in 83-91% of cases [2,3]. Other reported causes include use of anticoagulants, hemodialysis, aneurysmal bleeding and penetrating injuries [1]. ISH occur most often between the 6^th^ and 8^th^ decade and men are more often affected than women (male-to-female ratio 1.87:1) [3]. Patients often present with impairment of consciousness or contralateral hemiparesis with predominant involvement of the lower limb due to the physical representation of the primary motor cortex (homunculus). Other symptoms can include focal or generalized seizures, cognitive impairment, gait disorders and oculomotor nerve palsy [3].

## Case presentation

**Figure 1.**
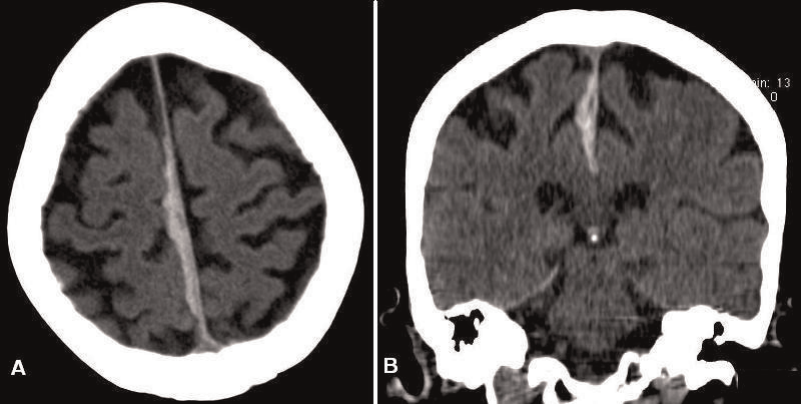
**(A,B)** Computed tomography of the brain on admission shows a 5 mm thick linear hyperdense lesion along the right side of the interhemispheric fissure.

**Figure 2.**
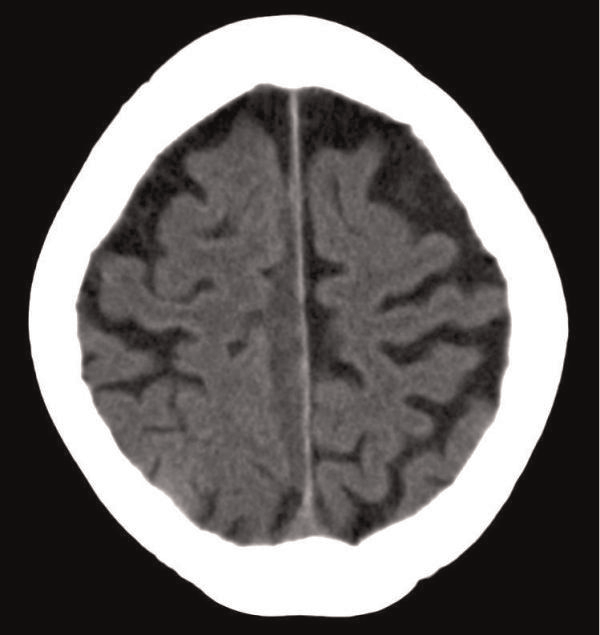
Computed tomography of the brain two weeks later shows a hypodense lesion representing an interhemispheric subdural hematoma of older age.

A 77-year-old Dutch Caucasian woman presented with a 2-day history of slight headache and progressive walking problems. No recent head trauma had occurred. Past medical history was extended including diabetes, ischemic stroke, hypertension and heart failure. She had been using among others an oral anticoagulant agent because of atrial fibrillation and mitral valve replacement. Neurological examination was unremarkable except for a disturbed heel to shin coordination test and an inability to walk without support. Results of laboratory studies revealed no significant abnormalities and an International Normalized Ratio of 2.6. Computed tomography of the brain showed a 5 mm thick linear hyperdense lesion extending along the right side of the interhemispheric fissure (Figure 1A and B). Because of the stable neurological condition the patient was admitted for observation and treated conservatively. The anticoagulant agent was stopped and gradual improvement was noticed in a few days. Five days after admission she was able to walk without assistance and she was discharged from the hospital. Two weeks later the patient was in excellent condition. Computed tomography of the brain revealed no signs of a rebleed and showed a hypodense lesion representing the known interhemispheric subdural hematoma of older age (Figure 2).

## Discussion

ISH are uncommon lesions and are usually seen after trauma or in patients with bleeding disorders (e.g., use of anticoagulants) [1]. Clinical presentation of patients with ISH can vary extensively which can make diagnosis difficult, especially in the absence of risk factors [3]. In this case, ataxia of both legs was the only abnormality revealed by neurologic examination. Due to the physical representation of the primary motor cortex, clinical symptoms in interhemispheric hematomas often predominantly involve the lower limbs, which can primarily manifest itself in ataxia. Depending on the severity of symptoms (e.g., level of consciousness and severity of neurological abnormalities) treatment has to be decided between conservative management or surgical intervention [4]. Whereas conservative treatment may be followed in those who are neurologically stable, surgical intervention is often reserved for patients with pronounced symptoms or neurological deficits [1]. The overall mortality rate of ISH ranges from 24-27%, independently of choice of treatment, but may increase up to 32% in patients with long-term altered levels of consciousness [3,5].

## Conclusion

Although ISH are uncommon, symptoms can be severe and clinical course can be devastating. Therefore, it is important that clinicians are aware of this condition, especially in patients with bleeding disorders (e.g., use of anticoagulants) or after head trauma.

## Abbreviation

ISH: interhemispheric subdural hematomas

## References

[bib-001] Shankar A, Joseph M, Chandy MJ (2003). Interhemispheric subdural hematoma: an uncommon sequel of trauma. Neurol India.

[bib-002] Sadrolhefazi A, Bloomfield SM (2000). Interhemispheric and bilateral chronic subdural hematoma. Neurosurg Clin N Am.

[bib-003] Bartels RH, Verhagen WI, Prick MJ, Dalman JE (1995). Interhemispheric subdural hematoma in adults: case reports and a review of the literature. Neurosurgery.

[bib-004] Furui T, Iwata K, Usui K (1988). Interhemispheric subdural haematoma complicated by intracerebral haematoma: case report. Acta Neurochir (Wien).

[bib-005] Borzone M, Altomonte M, Baldini M (1995). Typical interhemispheric subdural haematomas and falx syndrome: four cases and a review of the literature. Zentralbl Neurochir.

